# The Treatment of Certain Forms of Vomiting

**Published:** 1887-03

**Authors:** 


### *THE TREATMENT OF CERTAIN FORMS OF VOMITING.*

There are few disorders which cause more discomfort and distress than those accompanied with incessant attacks of vomiting; there are few disorders which try more the patience and the skill of the practitioner. Dr. F. P. Atkinson gives us in the *Practitioner* (for November, 1886,) a number of points which may prove useful in relieving certain forms of obstinate vomiting.

In cases of *simple bilious vomiting* he states that a mixture containing fifteen minims of solution of potassium and four of laudanum administered every four hours acts like a charm, and he asserts that in no uncomplicated cases will there be any vomiting after two or three doses. For the *vomiting of pregnancy* he suggested a little milk and tea, with a small piece of bread and butter or biscuit, immediately before rising in the morning, and a biscuit or two at various intervals throughout the day, whenever there is a feeling of emptiness In vomiting from *ulceration of the stomach* the great object is to give the stomach as much rest as possible. This may be accomplished by giving very small quantities of peptonized milk or koumiss at short intervals; thus a teaspoonful of the above may be mixed with cold water, and given every four hours. Dr. Atkinson further recommends that the body should be oiled night and morning to help nutrition, and covered with warm clothing to prevent cold. Later on, when the pain has almost subsided, various simple foods may be allowed. When the vomiting is very urgent, of course the stomach should be given entire rest, and peptonized meat enemata should be administered.

In the vomiting which occurs in *infants brought up by hand*, the most frequent cause is found in the inability to digest the casein of the milk. In such cases it is advisable to use one of the many peptonizing powders now on the market, or the following may be given: Two tablespoonfuls of whey, two tablespoonfuls of water, and one tablespoonful of cream; if there be some diarrhœa, a little meat juice may be given three or four times daily, while the body should be oiled night and morning.

